# Body Weight as a Determining Factor in the Predominance of Adverse Drug Reactions Induced by Fixed-Dose Adalimumab Injections in Female Patients in a Korean Hospital Setting

**DOI:** 10.3390/jcm9020461

**Published:** 2020-02-07

**Authors:** Kwi Suk Kim, Young Hee Choi, Aree Moon, Sang Geon Kim

**Affiliations:** 1 Department of Pharmacy, Seoul National University Hospital, Seoul 03080, Korea; ag0902@hanmail.net; 2 College of Pharmacy, Dongguk University Seoul, Goyang-si, Gyeonggi-do 10326, Korea; choiyh@dongguk.edu; 3 Duksung Innovative Drug Center, College of Pharmacy, Duksung Women’s University, Seoul 01369, Korea; armoon@duksung.ac.kr; 4 College of Pharmacy and Research Institute of Pharmaceutical Sciences, Seoul National University, 1 Gwanak-ro, Gwanak-gu, Seoul 08826, Korea

**Keywords:** adalimumab, adverse drug reaction, gender, body weight, skin, infection, test abnormality

## Abstract

Adalimumab is used at 40-mg dose to treat systemic inflammatory diseases. Given the impact of adverse drug reactions (ADRs), which particularly result in the discontinuation of adalimumab therapy in female patients, this study examined whether sex affects the frequency and type of ADRs induced by adalimumab. In this study, the prescription records and laboratory data of patients aged ≥19 years who had been admitted to the Seoul National University Hospital (SNUH) and prescribed adalimumab were analyzed using an electronic medical record database. The analysis revealed that female patients more frequently experienced adalimumab-induced ADRs compared with male patients (63.2% vs. 52.2%). The incidence of ADRs was significantly higher in female patients with ankylosing spondylitis or rheumatoid arthritis than in male patients with similar conditions (81.5% vs. 60.7% or 64.4% vs. 50.0%, respectively). The median body weight (BW) was lower in female patients than in male patients (54.0 vs. 66.0 kg). Moreover, the incidence of ADRs in patients with a BW of <54.0 kg (i.e., the median female BW) was higher than for those with a BW of ≥54.0 kg, in both males and females. Our results suggested that the predominance of ADRs induced by adalimumab in females was because of their relatively lower BW. This suggests the importance of BW as a determining factor in sex disparity of ADR occurrences.

## 1. Introduction

Adalimumab binds to tumor necrosis factor (TNF)-alpha and blocks interactions with the p55 and p75 cell surface TNF receptors, inhibiting inflammatory responses. The drug is widely prescribed for both male and female patients with rheumatoid arthritis, ankylosing spondylitis, Crohn’s disease, ulcerative colitis, and psoriasis [1,2], and also for patients with hidradenitis suppurativa, non-radiographic axial spondyloarthritis, peripheral spondyloarthritis, and non-infectious uveitis [3]. Adalimumab is currently marketed in injection vials and pens containing a fixed dose of 40 mg for adults, regardless of differences in sex or body weight (BW) [4]. The common side effects of adalimumab include injection site pain, upper respiratory tract infection, increased creatine phosphokinase levels, headache, rash, sinusitis, nausea, urinary tract infection, abdominal pain, flu-like syndrome, hyperlipidemia, back pain, high cholesterol, hemuresis, and hypertension. The occurrences and severity of adverse drug reactions (ADRs) vary among patients treated with adalimumab [5,6,7]; however, the exact reasons for these variations have remained elusive.

It has been recognized that the frequent and common adalimumab-induced ADRs have resulted in a fear of such deleterious effects, leading to a decrease in drug compliance [5,6,7,8]. Indeed, there have been controversial reports of sex-dependent ADRs induced by adalimumab therapy. A small group of prospective clinical cohort studies showed that adalimumab induced more ADRs in female patients, who more often discontinued the drug owing to negative effects [8]. In another study, female sex was considered to be the strongest predictor of higher anti-TNF drug discontinuation rates [9,10]. Moreover, it has been reported that female sex is associated with lower trough levels of adalimumab and a higher incidence of adalimumab antibody production [11]. Additionally, a prospective, comparative, multicenter, and long-term drug-survival study has indicated that a higher body mass index (BMI) is a predictor of discontinuation of certain biological agents, including adalimumab, owing to their adverse effects or inefficacy [12,13]. Thus, female sex has been reported as a consistent predictor for drug discontinuation owing to side effects.

It has been claimed that differences between the two sexes regarding body composition, such as smaller organ sizes and higher proportion of body fat in females, may result in different pharmacokinetics of certain biological agents (e.g., rituximab and cetuximab) [14,15]. These differences, along with hormonal differences, suggest the requirement of sex-dependent dosage regimens. Hence, further analysis may be necessary to understand the basis of ADRs with regard to sex differences. Given the sex-skewed impact of ADRs on the discontinuation of adalimumab therapy and our preliminary analysis of spontaneous ADR incidence reports over the past decade, we aimed to examine whether there were any sex-based differences in ADRs induced in patients receiving adalimumab medications using an electronic medical record (EMR) database, in conjunction with test results and nursing records, in Seoul National University Hospital (SNUH) between 2008 and 2018. In this study, we proposed that an average BW of 54.0 kg was the threshold that determined ADR types and adalimumab frequencies and that the predominance of ADRs in female patients may result from the fixed-dose injection regimens administered to adults, regardless of an individual patient’s BW.

## 2. Experimental Section

### 2.1. EMR Data Collection

The study was approved by the Institutional Review Board of SNUH (IRB# H-1803-095-930), which is a 1779-bed medical center in the Republic of Korea. The records of 792 patients aged ≥19 years who were admitted to SNUH and had been prescribed adalimumab at least once between February 2008 and May 2018 were extracted from the EMR database. After the records of patients with cancelled prescriptions of adalimumab (*n* = 65) were removed, 727 patient records remained. The collected data contained anonymous codes representing patient files comprising age, sex, height, initial BW, medical diagnosis codes, concomitant diseases, ADRs, dates of laboratory tests conducted and medications (generic and brand name), and prescription date and duration.

### 2.2. Analysis of Prescription and Laboratory Data

On the basis of the laboratory data, various parameters, including diseases, prescription information for adalimumab (e.g., prescription period and co-prescribed drugs), types and frequencies of ADRs, and modification of prescriptions after ADRs, were investigated.

### 2.3. Statistics

All values are presented as medians (ranges) or averages ± standard deviations, as appropriate. The chi-square test was performed to assess any sex-based differences in the occurrence of ADRs. Statistical analyses were computed by using the IBM SPSS statistics 23.0 software package (SPSS Inc., Chicago, IL, USA).

## 3. Results

### 3.1. Patient Information and Evaluation of Adalimumab Efficacy

**Figure 1:**
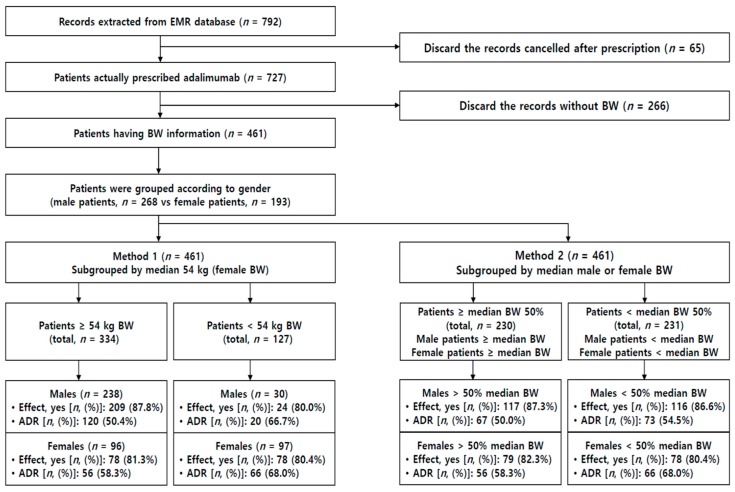
A flowchart showing subgrouping of patients, adverse drug reactions (ADRs), adalimumab in patients with records of body weight (BW).

Patients who were prescribed adalimumab were divided into subgroups according to BW cutoffs and sex as shown in Figure 1. The demographic characteristics of all patients who were administered adalimumab are summarized in Table S1, which shows a significantly higher incidence of ADRs in female patients than in male patients (60.9% vs. 50.9%, *p* < 0.001). In our analysis, adalimumab was prescribed to patients with various conditions, such as ankylosing spondylitis (A); Bechet’s disease (B); Crohn’s disease (C); Bechet’s disease plus Crohn’s disease (B + C); psoriasis (P); rheumatoid arthritis (R); and comorbid diseases. Most subgroups of female patients with each of these diseases had a significantly higher incidence of ADRs than the corresponding subgroups of male patients (Table S1). The efficacy of adalimumab was comparable between both the sexes. In addition, there was a significant sex-related difference in age among the patients treated with adalimumab (*n* = 727) (Table S1): Female patients were significantly older than male patients (46.0 vs. 34.5 years, *p* < 0.001).

The analysis of patients’ BW data (*n* = 461) revealed a notable difference in mean BW between male and female patients (66.0 vs. 54.0 kg, *p* = 0.038; Table 1), suggesting that BW is a discriminating factor. In addition, the mean age of female patients was higher than that of male patients (48.0 vs. 35.5 years, *p* < 0.001).

Compared with female patients, male patients were more frequently treated for ankylosing spondylitis, Bechet’s disease, and Crohn’s disease among all conditions. Conversely, treatments for rheumatoid arthritis were more frequently administered to female patients (Table 1). The responders to adalimumab were marked as “efficient” or “improving laboratory results,” whereas the non-responders were marked as “inefficient,” “stopped administration,” or “changed to another biologic” from the reviews of clinical signs or laboratory data in the EMR data files. To assess disease progression, the efficacy of adalimumab was evaluated every 3 months in accordance with the guidelines of health insurance benefits. Sex-based differences were not observed in the overall efficacy of adalimumab for the studied conditions (i.e., 86.9% in male patients vs. 83.6% in female patients) (Table 1). The subgroup analyses for the patients with different diagnoses revealed no significant differences in efficacy between the sexes (Table 1).

### 3.2. ADR Incidence Rates

As the same fixed dose of adalimumab (40 mg) was subcutaneously administered to all patients [3], we investigated if BW may be the causative factor of the higher incidence rate of ADRs in women. To identify the factors that determined the ADRs induced by adalimumab, we examined the profile and incidence rates of ADRs by reviewing electronic charts and laboratory results, with particular attention to sex-related changes in ADR frequency in populations with different tiers of BW (*n* = 461) (Table 1). As expected, the overall incidence rate of ADRs was significantly higher in women than in men (63.2% vs. 52.2%, *p* = 0.019) (Table 1). The average number of ADRs per patient (1.6) was the same for male and female patients as shown in Table 1. In addition, of all the conditions treated by adalimumab, the incidence of ADRs was significantly higher in female patients with ankylosing spondylitis than in corresponding male patients (81.5% vs. 60.7%, *p* = 0.041) (Table 1). There were no statistically significant differences in other disease categories. For all listed conditions, there were no sex-based differences in pharmacological effects.

For patients with a record of initial BW while starting adalimumab prescription therapy, the incidence of ADRs was compared with regard to different BW subgroups (Method 2 in Figure 1). When we compared the ADR frequencies in the subgroup of the patients with lower 50% of BW, the proportion of patients experiencing ADRs was greater in female patients than in male patients (i.e., 68.0% vs. 54.5%, *p* = 0.038). However, there was no sex-related difference in the subgroup of the patients with upper 50% of BW (58.3% vs. 50.0%, *p* = 0.211) (Table S2).

According to Method 1 in Figure 1, when we used the median female BW (54.0 kg) as a cutoff value, the incidence of ADRs was significantly different between male and female patients with BW ≥ 54.0 kg (50.4% vs. 58.3%, *p* < 0.001), and the incidence of ADRs was similar in male and female patients with BW < 54.0 kg (66.7% vs. 68.0%, *p* = 0.888) (Table 2). However, for both the sexes, ADR incidence in patients with BW < 54.0 kg was significantly higher than that in patients with BW ≥ 54.0 kg (66.7% vs. 50.4%, *p* = 0.068 in male patients; and 68.0% vs. 58.3%, *p* = 0.162 in female patients) (Table 2). Thus, both male and female patients with BW lower than the median female BW displayed a greater incidence of ADRs. Therefore, it is highly likely that the predominance of ADRs in female patients may result from their relatively lower BW, indicating the importance of BW as a determining factor of ADR occurrences.

**Figure 2:**
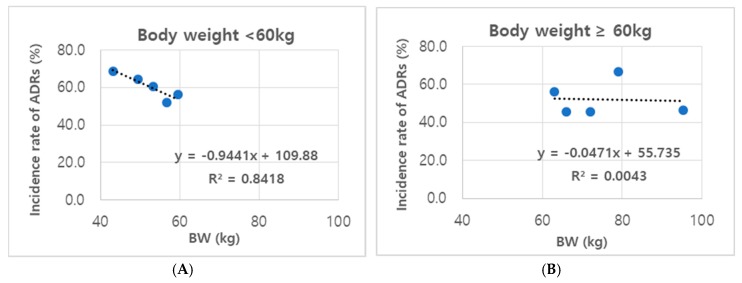
ADR incidence rates in subgroups tiered by BW. (**A**) Male and female patients with a BW < 60.0 kg and (**B**) Male and female patients with a BW ≥ 60.0 kg.

The median BW of all patients examined in this study was approximately 60.0 kg. When we compared ADR frequencies in the subgroups of patients with different BW, we found that subgroups of both male and female patients with a BW < 60.0 kg showed BW-dependent frequencies of ADR, with an inverse correlation (a = −0.9441, r^2^ = 0.8418) (Figure 2A). The correlation between ADR frequency and BW did not exist in patients with BW ≥ 60.0 kg (a = −0.0471, r^2^ = 0.0043) (Figure 2B). Thus, it appears that both male and female patients with a BW of 60.0 kg may be more vulnerable to ADRs induced by adalimumab injections.

### 3.3. ADR Types

The ADRs of adalimumab identified in the present study included infections (i.e., uvenitis, rhinitis, pneumonia, tuberculosis, virus infection, and fungus infection), pain, abnormal lab tests, mental/mood changes, and skin and gastrointestinal tract problems. Infections and dermatological problems were more prevalent than other ADRs (Table 3). A significantly higher frequency of skin problems and insomnia (psychiatrics) was noted in female patients than in male patients (31.1% vs. 22.0%, *p* = 0.03 and 5.0% vs. 1.5%, *p* = 0.012, respectively). In particular, more female patients with ankylosing spondylitis reported skin problems (Table 3). Among patients with Bechet’s disease or Crohn’s disease, abnormal lab tests were more common in female patients (Table 3).

## 4. Discussion

Recognizing ADRs has emerged as a key factor in deciding an appropriate medication therapy for a particular condition. As adverse reactions and toxicities of drugs may vary depending on sex, information on ADRs should provide incidence rates, severity, durations, and onset times of parameters of interest in both the sexes. ADRs induced by drugs may vary between the sexes owing to differences in height, weight, pharmacokinetics, and genetic characteristics [16,17]. However, many previous studies mostly have included men [18,19]. In general, the medication guidelines, systems, and policies that specify sex differences appear to be incomplete. We first examined a 10-year period of voluntary ADR reports archived in the Korean Institute of Drug Safety and Risk Management and extracted drug items that led to potential differences in the occurrence of ADRs based on sex. However, as these archives included only voluntary reports, it was not plausible to estimate the overall proportion and incidence rate. Thus, the present study utilized an EMR database to analyze the incidence, type, and severity of ADRs induced by adalimumab.

Adalimumab is administered to adults as a 40-mg solution for injection in a pre-filled syringe. In the context of patient BW, the dose is only adjusted for patients with polyarticular juvenile idiopathic arthritis or with plaque psoriasis. The recommended dosing regimen is 20 mg every other week for patients with a BW of 10.0 kg to <30.0 kg and 40 mg every other week for those with a BW of ≥30.0 kg. The available data suggest that clinical response is usually achieved within 12 weeks of treatment [20]. If patients do not exhibit a response during this period, continued therapy should be reconsidered [21]. The dose of adalimumab for pediatric patients with Crohn’s disease is also based on BW. The induction dose is 80 mg during week 0 and 40 mg during week 2 for patients with a BW of <40.0 kg, if there is a need for a more rapid response to therapy, and with the awareness that the risk of adverse events may be higher with use of a higher induction dose (a maintenance dose of 20 mg every other week is recommended). For patients with a BW of ≥40.0 kg, the doses are doubled (i.e., 160 mg during week 0 and 80 mg during week 2) [22]. Hence, the regular dose of adalimumab for adults with inflammatory diseases is usually 40 mg per patient, regardless of the sex and BW. The prescription rate of adalimumab has gradually increased since its introduction in the market [22,23], and there will be several more adalimumab biosimilars available in the future [23,24].

In the study of Lesuis et al. [25], the proportion of rheumatoid arthritis in patients was higher in women and rates of inflammatory bowel disease and psoriasis were higher in men [25]. In the present study, rheumatoid arthritis was also more common in women but rates of inflammatory bowel disease and psoriasis were similar in men and women. In other studies, female sex has been reported as a predictor for biological discontinuation of adalimumab in the treatment of psoriasis [26], rheumatoid arthritis, and axial spondyloarthritis [27]. The incidence of ADRs induced by adalimumab was greater in female patients, although the types of adverse reactions were different [28]. Therefore, among the non-responders, the discontinuation rate of adalimumab was higher in female patients [28,29].

An important finding of our study was the identification of BW as a factor that determines the predominance of ADRs in female patients. This idea was supported by the different frequencies of ADRs in the analysis of subgroups based on BW tiers in male and female patients. The frequency of ADRs in either male or female patients with a BW of <60.0 kg exhibited BW dependency likely because the median BW was significantly lower in female patients than in male patients (the proportion of female patients with a BW of <60.0 kg was 68.0%). Additionally, Hansel et al. [30] demonstrated the importance of BMI in determining a dosage regimen of adalimumab. Dose tapering with adalimumab was efficacious and safe, especially in patients with low BMI. These patients showed maintenance of efficacy and, in cases of relapse, only needed a short time to re-achieve disease clearance at a standard dose [30].

The incidence and characteristics of ADRs in the present study were similar to those in a study by Noah et al. [29]: 28.4% patients in our study experienced dermatological problems (Table 3), which was similar to the 30% of patients with skin and allergenic reactions in the study by Noah et al. [31]. In another study, the percentage of ADRs in patients with rheumatoid arthritis followed for 3 years after the start of adalimumab treatment was 34.2% [32]. In our study, the frequency of ADRs induced by adalimumab used to treat rheumatoid arthritis was 60.0% (19 male patients and 56 female patients among a total of 125 patients) (Table 1). The difference might be attributed to our prolonged follow-up period (5.5 years). Adalimumab dose adjustment is usually done by controlling either the first dose or the maintenance dose and the dosing intervals [33]. After considering the long half-life of this drug, extending dosing intervals might be recommended for female patients to reduce ADRs because higher concentration of adalimumab might cause higher incidence of ADRs. As skin-related ADRs and insomnia were more prevalent in female patients, it is also necessary to monitor and educate the patients on these sex-based differences.

## 5. Conclusions

Collectively, our findings indicate that the incidence of ADRs induced by adalimumab therapy was higher in female patients, which may have been because of the difference in mean BW (i.e., a greater proportion of female patients had a lower BW) and the features of adalimumab injectable medication. This was consistent with the reported increase in the incidence of ADRs induced by adalimumab with a decrease in BW, which occurs owing to increasing adalimumab concentrations due to the reduced elimination rate [34,35].

## Figures and Tables

**Table 1:** The efficacy and adverse drug reactions (ADRs) of adalimumab in patients with records of body weight (BW).

Categories			
Total (*n* = 461)	Male (*n* = 268)	Female (*n* = 193)	*p*-Values
Age (Median, (min-max)) *	35.5 (19–78)	48 (19–84)	*p* < 0.001
BW (Median, (min-max)) *	66.0 (36.8–120)	54.0 (31.7–80.0)	*p* = 0.038
Effect (*n*, (%))			
Yes	233 (86.9%)	156 (83.6%)	*p* = 0.075
No *	25 (9.3%)	32 (13.5%)	*p* = 0.054
Non-Judgment	7 (2.6%)	5 (2.9%)	*p* = 0.989
Number of Patients with ADRs (*n*, (%)) *	140 (52.2%)	122 (63.2%)	*p* = 0.019
Number of ADRs (*n*)	223	199	*p* = 0.155
ADR per Patient (No. of Incidences/No. of Patients with ADR)	1.6	1.6	
Diagnosis			
(1) Ankylosing Spondylitis (A)			
Total (*n* = 149)	Male (*n* = 122)	Female (*n* = 27)	
Age (Median, (min-max))	36 (19–78)	41 (19–67)	*p* = 0.401
BW (Median, (min-max))	72.0 (50.0–120.0)	58.4 (38.6–80.0)	*p* = 0.152
Effect (*n*, (%))	109 (89.3%)	22 (81.5%)	*p* = 0.257
Number of Patients with ADRs (*n*, (%)) *	74 (60.7%)	22 (81.5%)	*p* = 0.041
Number of ADRs (*n*)	122	43	*p* = 0.066
ADR Cases per Patient	1.6	2.0	
(2) Rheumatoid Arthritis (R)			
Total (*n* = 125)	Male (*n* = 38)	Female (*n* = 87)	
Age (Median, (min-max))	31.5 (19–78)	54 (19–84)	*p* = 0.194
BW (Median, (min-max))	65.0 (36.8–100.0)	55.0 (31.7–74.0)	*p* = 0.626
Effect (*n*, (%))	33 (86.8%)	67 (77.0%)	*p* = 0.206
Number of Patients with ADRs (*n*, (%))	19 (50.0%)	56 (64.4%)	*p* = 0.131
Number of ADRs (*n*) *	36	79	*p* = 0.037
ADR Cases per Patient	1.9	1.4	
(3) Bechet’s Disease Plus Crohn’s Disease (B + C)			
Total (*n* = 144)	Male (*n* = 91)	Female (*n* = 53)	
Age (Median, (min-max))	35 (19–71)	36 (19–75)	*p* = 0.093
BW (Median, (min-max))	60.3 (38.5–98.6)	49.6 (38.3–76.6)	*p* = 0.241
Effect (*n*, (%))	76 (83.5%)	45 (84.9%)	*p* = 0.826
Number of Patients with ADRs (*n*, (%))	37 (40.7%)	30 (56.6%)	*p* = 0.064
Number of ADRs (*n*)	51	53	*p* = 0.115
ADR Cases per Patient	1.4	1.8	
(4) Psoriasis (P)			
Total (*n* = 15)	Male (*n* = 8)	Female (*n* = 7)	
Age (Median, (min-max))	38 (25–67)	58 (46–64)	*p* = 0.307
BW (Median, (min-max))	71.5 (56.7–87.0)	54.0 (48.2–65.0)	*p* = 0.449
Effect (*n*, (%))	6 (75.0%)	6 (85.7%)	*p* = 0.605
Number of Patient with ADRs (*n*, (%))	5 (62.5%)	5 (71.4%)	*p* = 0.714
Number of ADRs (*n*)	6	10	*p* = 0.267
ADR Cases per Patient	1.2	2.0	
(5) Two or More Other Diseases (Comorbid Diseases)			
Total (*n* = 17)	Male (*n* = 6)	Female (*n* = 11)	
Age (Median, (min-max))	47 (22–60)	37 (19–71)	*p* = 0.319
BW (Median, (min-max))	57.9 (54.0–86.0)	57.0 (34.6–63.2)	*p* = 0.386
Effect (*n*, (%))	6 (100.0%)	9 (81.8%)	*p* = 0.266
Number of Patients with ADRs (*n*, (%))	5 (83.3%)	7 (63.6%)	*p* = 0.394
Number of ADRs (*n*)	8	11	*p* = 0.315
ADR Cases per Patient	1.6	1.6	
(6) Others (E)			
Total (*n* = 11)	Male (*n* = 3)	Female (*n* = 8)	
Age (Median, (min-max))	36 (36–48)	26.5 (19–71)	*p* = 0.388
BW [median, (min-max))	95.7 (86.8–109.2)	54.1 (49.0–74.5)	*p* = 0.358
Effect (*n*, (%))	3 (100.0%)	7 (87.5%)	*p* = 0.521
Number of Patients with ADRs (*n*, (%))	0 (0.0%)	2 (25.0%)	*p* = 0.338
Number of ADRs (*n*)	0	3	*p* = 0.632
ADR Cases Per Patient	0.0	1.5	

* Items marked are statistically significant for gender differences.

**Table 2:** ADR incidence rates in subgroups divided by the cutoff of female median body weight (BW) (54 kg).

	Males	Females	*p*-Values
BW ≥ 54.0 kg (*n* = 334)			
Number of Patients (*n*, (%))	238 (71.3%)	96 (28.7%)	
Age (Median, (min–max)) *	36 (19–78)	50.5 (19–84)	*p* = 0.028
BW (Median, (min-max))	67.5 (54.0–120.0)	59.8 (54.0–80.0)	*p* = 0.215
Effect, Yes (*n*, (%))	209 (87.8%)	78 (81.3%)	*p* = 0.118
Number of Patients with ADRs (*n*, (%)) *	120 (50.4%)	56 (58.3%)	*p* < 0.001
ADR Cases per Patient (Incidences/Number of Patients with ADR)	1.56	1.66	*p* = 0.470
BW < 54.0 kg (*n* = 127)			
Number of Patients (*n*, (%))	30 (23.6%)	97 (76.4%)	
Age (Median, (min-max))	30.5 (20–78)	47 (19–79)	*p* = 0.271
BW (Median, (min-max))	49.7 (36.8–53.5)	49.0 (31.7–53.9)	*p* = 0.358
Effect, Yes (*n*, (%))	24 (80.0%)	78 (80.4%)	*p* = 0.960
Number of Patients with ADRs (*n*, (%))	20 (66.7%)	66 (68.0%)	*p* = 0.888
ADR Cases per Patient (Incidences/Number of Patients with ADR)	1.75	1.61	*p* = 0.586

* Items marked are statistically significant for gender differences.

**Table 3:** Types of ADRs in male or female patients with different diseases.

Types of ADRs		Male	Female	*p*-Value
**Total Number of Patients (*n* = 461) (*n*, (%))**		268 (58.1%)	193 (41.9%)	
Infection (*n*, (%))		59 (22.0%)	44 (22.8%)	*p* = 0.768
Pain (*n*, (%))		18 (6.7%)	16 (8.3%)	*p* = 0.993
Dermatologicals (*n*, (%))	Skin *	59 (22.0%)	60 (31.1%)	*p* = 0.030
	Alopecia	5 (1.9%)	7 (3.6%)	*p* = 0.374
Gastrointestinals (*n*, (%))		26 (9.7%)	17 (8.8%)	*p* = 0.473
Psychiatrics (*n*, (%)) *		4 (1.5%)	11 (5.0%)	*p* = 0.012
Abnormal Lab Test (*n*, (%))		32 (12.0%)	30 (15.6%)	*p* = 0.625
Others (*n*, (%))		19 (7.1%)	14 (7.3%)	*p* = 0.946
(1) Ankylosing Spondylitis (A)				
Number of Patients (*n* = 149) (*n*, (%))		122 (81.9%)	27 (18.1%)	
Infection (*n*, (%))		33 (27.0%)	8 (29.6%)	*p* = 0.786
Pain (*n*, (%))		9 (7.4%)	5 (18.5%)	*p* = 0.073
Dermatologicals (*n*, (%))	Skin *	32 (26.2%)	13 (48.1%)	*p* = 0.025
	Alopecia	2 (1.6%)	0 (0.0%)	*p* = 0.503
Gastrointestinals (*n*, (%))		20 (16.4%)	3 (11.1%)	*p* = 0.492
Psychiatrics (*n*, (%))		4 (3.3%)	3 (1.6%)	*p* = 0.082
Abnormal Lab Test (*n*, (%))		17 (14.0%)	7 (25.9%)	*p* = 0.125
Others (*n*, (%)) *		5 (4.1%)	4 (14.8%)	*p* = 0.034
(2) Rheumatoid Arthritis (R)				
Number of Patients (*n* = 125) (*n*, (%))		38 (30.4%)	87 (69.6%)	
Infection (*n*, (%))		9 (23.7%)	19 (21.8%)	*p* = 0.820
Pain (*n*, (%))		4 (10.5%)	7 (8.0%)	*p* = 0.653
Dermatologicals (*n*, (%))	Skin	8 (21.1%)	22 (25.3%)	*p* = 0.481
	Alopecia	1 (2.6%)	4 (4.6%)	*p* = 0.873
Gastrointestinals (*n*, (%))		4 (10.5%)	8 (9.2%)	*p* = 0.816
Psychiatrics (*n*, (%))		0 (0.0%)	5 (1.1%)	*p* = 0.131
Abnormal Lab Test (*n*, (%))		3 (7.9%)	6 (6.8%)	*p* = 0.843
Others (*n*, (%))		6 (15.8%)	8 (9.2%)	*p* = 0.389
(3) Bechet’s Disease Plus Crohn’s Disease (B + C)				
Subtotal (*n* = 144) (*n*, (%))		91 (63.2%)	53 (43.8%)	
Infection (*n*, (%))		14 (15.4%)	11 (20.8%)	*p* = 0.412
Pain (*n*, (%))		4 (4.4%)	3 (5.7%)	*p* = 0.426
Dermatologicals (*n*, (%))	Skin	15 (16.5%)	15 (28.3%)	*p* = 0.092
	Alopecia	1 (1.1%)	3 (5.7%)	*p* = 0.108
Gastrointestinals (*n*, (%))		2 (2.2%)	3 (5.7%)	*p* = 0.274
Psychiatrics (*n*, (%))		0 (0.0%)	2 (3.8%)	*p* = 0.062
Abnormal Lab Test (*n*, (%)) *		10 (11.0%)	14 (26.4%)	*p* = 0.017
Others (*n*, (%))		5 (5.5%)	1 (1.9%)	*p* = 0.296
(4) Psoriasis (P)				
Subtotal (*n* = 15) (*n*, (%))		8 (53.3%)	7 (46.7%)	
Infection (n, (%))		2 (25.0%)	2 (28.6%)	*p* = 0.876
Pain (*n*, (%))		0 (0.0%)	0 (0.0%)	-
Dermatologicals (*n*, (%))	Skin	1 (12.5%)	3 (42.9%)	*p* = 0.185
	Alopecia	1 (12.5%)	0 (0.0%)	*p* = 0.333
Gastrointestinals (*n*, (%))		0 (0.0%)	2 (28.6%)	*p* = 0.104
Psychiatrics (*n*, (%))		0 (0.0%)	1 (14.3%)	*p* = 0.268
Abnormal Lab Test (*n*, (%))		1 (12.5%)	2 (28.6%)	*p* = 0.438
Others (*n*, (%))		1 (12.5%)	0 (0.0%)	*p* = 0.333
(5) Two or More Other Diseases (Comorbid Diseases)				
Subtotal (*n* = 17) (*n*, (%))		6 (35.3%)	11 (64.7%)	
Infection (*n*, (%))		1 (16.7%)	3 (27.3%)	*p* = 0.159
Pain (*n*, (%))		1 (16.7%)	1 (9.1%)	*p* = 0.643
Dermatologicals (*n*, (%))	Skin	3 (50.0%)	7 (63.6%)	*p* = 0.585
	Alopecia	0 (0.0%)	0 (0.0%)	-
Gastrointestinals (*n*, (%))		0 (0.0%)	0 (0.0%)	-
Psychiatrics (*n*, (%))		0 (0.0%)	0 (0.0%)	-
Abnormal Lab Test (*n*, %)		1 (16.7%)	1 (9.1%)	*p* = 0.643
Others (*n*, (%)) *		2 (33.3%)	0 (0.0%)	*p* = 0.041
(6) Others (E)				
Subtotal (*n* = 11) (*n*, (%))		3 (27.3%)	8 (72.7%)	
Infection (*n*, (%))		0 (0.0%)	1 (12.5%)	*p* = 0.521
Pain (*n*, (%))		0 (0.0%)	0 (0.0%)	-
Dermatologicals (*n*, (%))	Skin	0 (0.0%)	0 (0.0%)	-
	Alopecia	0 (0.0%)	0 (0.0%)	-
Gastrointestinals (*n*, (%))		0 (0.0%)	1 (12.5%)	*p* = 0.521
Psychiatrics (*n*, (%))		0 (0.0%)	0 (0.0%)	-
Abnormal Lab Test (*n*, (%))		0 (0.0%)	0 (0.0%)	-
Others (*n*, (%))		0 (0.0%)	1 (12.5%)	*p* = 0.521

* Items marked are statistically significant for gender differences.

## Supplementary Materials

The following are available online at https://www.mdpi.com/2077-0383/9/2/461/s1, Table S1: Demographic characteristics of all patients administered adalimumab; Table S2: Effectiveness and ADR incidences in male or female patients sub-grouped by 50 percentile.
