# Supplementary material for: Body Weight as a Determining Factor in the Predominance of Adverse Drug Reactions Induced by Fixed-Dose Adalimumab Injections in Female Patients in a Korean Hospital Setting
Source: J Clin Med. 2020 Feb 7;9(2):461. doi: 10.3390/jcm9020461 (PMC7074186; doi:10.3390/jcm9020461)
Supplement: Supplementary file 1 [file jcm-09-00461-s001.pdf]

Supplementary Material

Body Weight as a Determining Factor in the Predominance of Adverse Drug Reactions Induced by Fixed-Dose Adalimumab Injections in Female Patients in a Korean Hospital Setting

Kwi Suk Kim [1], Young Hee Choi [2], Aree Moon [3] and Sang Geon Kim [1,4,*]

[1] Department of Pharmacy, Seoul National University Hospital, Seoul 03080, Korea; ag0902@hanmail.net

[2] College of Pharmacy, Dongguk University Seoul, Goyang-si, Gyeonggi-do 10326, Korea; choiyh@dongguk.edu

[3] Duksung Innovative Drug Center, College of Pharmacy, Duksung Women's University, Seoul 01369, Korea; armoon@duksung.ac.kr

[4] College of Pharmacy and Research Institute of Pharmaceutical Sciences, Seoul National University, 1 Gwanak-ro, Gwanak-gu, Seoul 08826, Korea

* Correspondence: sgk@snu.ac.kr; Tel.: +82-2-880-7840

Table 1. Demographic characteristics of all patients administered adalimumab.

**Total (n = 727)**	**Male (n = 446)**	**Female (n = 281)**	**p - values**
Age [Median, (min-max)]*	34.5 (19–78)	46 (19–84)	$p < 0.001$
Effect [n, (%)]			
Yes	394 (88.3%)	235 (83.6%)	$p = 0.120$
No	45 (10.1%)	38 (13.5%)	$p = 0.052$
Non-Judgment	7 (1.6%)	8 (2.9%)	$p = 0.238$
Number of Patients with ADRs [n, (%)]*	227 (50.9%)	171 (60.9%)	$p < 0.001$
Number of ADRs [n]	352	290	
ADR per Patient*	1.6	1.7	$p = 0.044$
Sub-Grouped by Diagnosis			
1) A			
Total (n = 320)	Male (n = 262)	Female (n = 58)	
Age [Median, (min-max)] *	33 (19–78) [a]	58 (19–67)	$p = 0.038$
Effect [n, (%)]	238 (90.8%)	53 (91.4%)	$p = 0.897$
Number of Patients with ADRs [n, (%)]*	148 (56.5%)	42 (72.4%)	$p < 0.001$
Number of ADRs [n]	229	85	
ADR Cases per Patient*	1.5	2.0	$p = 0.002$
2) R			
Total (n = 172)	Male (n = 46)	Female (n = 126)	
Age [Median, (min-max)]	36 (19–78)	52 (19–84)	$p = 0.158$
Effect [n, (%)]	39 (84.8%)	98 (77.8%)	$p = 0.312$
Number of Patients with ADRs [n, (%)]*	22 (47.8%)	76 (60.3%)	$p < 0.001$
Number of ADRs [n]	40	112	
ADR Cases per Patient	1.8	1.5	$p = 0.102$
3) B + C			
Total (n = 167)	Male (n = 105)	Female (n = 62)	

Age [Median, (min-max)]	36 (19–71)	36.5 (19–75)	$p = 0.061$
Effect [n, (%)]	87 (82.9%)	53 (86.9%)	P= 0.656
Number of Patients with ADRs [n, (%)]*	41 (39.0%)	32 (52.5%)	$p < 0.001$
Number of ADRs [n]	59	58	
ADR Cases per Patient	1.4	1.8	$p = 0.229$
4) P			
Total ($n = 31$)	Male ($n = 18$)	Female ($n = 13$)	
Age [Median, (min-max)]	38 (25–67)	54 (26–64)	$p = 0.309$
Effect [n, (%)]	16 (88.9%)	12 (92.3%)	$p = 0.751$
Number of Patient with ADRs [n, (%)]*	9 (50.0%)	10 (76.9%)	$p < 0.001$
Number of ADRs [n]	13	18	
ADR Cases per Patient	1.4	1.8	$p = 0.220$
5) Comorbid Disease			
Total ($n = 22$)	Male ($n = 10$)	Female ($n = 12$)	
Age [Median, (min-max)]	47 (19–60)	38 (19–71)	$p = 0.527$
Effect [n, (%)]	9 (90.0%)	10 (83.3%)	$p = 0.650$
Number of Patients with ADRs [n, (%)]*	7 (70.0%)	8 (66.7%)	$p < 0.001$
Number of ADRs [n]	11	12	
ADR Cases per Patient	1.6	1.5	$p = 0.678$
6) E			
Total ($n = 15$)	Male ($n = 5$)	Female ($n = 10$)	
Age [Median, (min-max)]	36 (23–48)	37.5 (19–71)	$p = 0.285$
Effect [n, (%)]	5 (100.0%)	9 (90.0%)	$p = 0.464$
Number of Patients with ADRs [n, (%)]*	0 (0.0%)	3 (30.0%)	$p < 0.001$
Number of ADRs [n]	0	4	
ADR Cases per Patient	0.0	1.3	$p = 0.392$

Abbreviations; A, Ankylosing spondylitis; B, Bechet's disease; C, Crohn's disease; P, Psoriasis; R, Rheumatoid arthritis; Complex, two or more diseases; and E, others

ADR cases per patient were calculated by counting ADR incidences divided by number of patients with ADR.

* Items marked are statistically significant between males and females.

Table S2. Effectiveness and ADR incidences in male or female patients sub-grouped by 50 percentile

		Males	Females	*p* - values
**Total (n = 461)**				
Subgrouped by Percentiles of Patients in Each Gender				
Patients with High 50 Percentile BW (n = 230)				
	Number of Patients [n, (%)]	134 (58.3%)	96 (41.7%)	
	Age [Median, (min-max)]*	35 (19–74)	50.5 (19–84)	$p = 0.036$
	Body Weight [Median, (min-max)]*	75.3 (66.0–120.0)	59.8 (54.0–80.0)	$p = 0.017$
	Effect, Yes [n, (%)]	117 (87.3%)	79 (82.3%)	$p = 0.290$
	Number of Patients with ADRs [n, (%)]	67 (50.0%)	56 (58.3%)	$p = 0.211$
	ADR Cases Per Patient (Incidences/Number of Patients with ADR)	1.6	1.7	$p = 0.508$
Patients with Low 50 Percentile BW (n = 231)				
	Number of Patients [n, (%)]	134 (58.0%)	97 (42.0%)	
	Age [Median, (min-max)]*	36 (19–78)	43 (19–79)	$p = 0.010$
	Body Weight [Median, (min-max)]*	59.0 (36.8–66.0)	49.0 (31.7-53.9)	$p = 0.007$
	Effect, Yes [n, (%)]	116 (86.6%)	78 (80.4%)	$p = 0.208$
	Number of Patients with ADRs [n, (%)]*	73 (54.5%)	66 (68.0%)	$p = 0.038$
	ADR Cases per Patient (Incidences/Number of Patients with ADR)	1.6	1.6	$p = 0.317$

*Items marked are statistically significant for gender differences.
